# Perioperative rifaximin is not associated with enhanced functional and volumetric recovery after major liver resection

**DOI:** 10.1038/s41598-021-97442-w

**Published:** 2021-09-09

**Authors:** Jan Bednarsch, Zoltan Czigany, Sven H. Loosen, Lara Heij, Lorenz Ruckgaber, Henning Maes, Jan-Pit Krause, Matthias Reen, Beata Toteva, Theresa Vosdellen, Philipp Bruners, Sven Arke Lang, Tom Florian Ulmer, Christoph Roderburg, Tom Luedde, Ulf Peter Neumann

**Affiliations:** 1 Department of Surgery and Transplantation, University Hospital RWTH Aachen, Pauwelsstrasse 30, 52074 Aachen, Germany; 2 Department of Medicine III, University Hospital RWTH Aachen, Aachen, Germany; 3 Department of Gastroenterology, Hepatology and Infectious Diseases, University Hospital Heinrich Heine University, Duesseldorf, Germany; 4 Institute of Pathology, University Hospital RWTH Aachen, Aachen, Germany; 5 Department of Radiology, University Hospital RWTH Aachen, Aachen, Germany; 6 Department of Surgery, Maastricht University Medical Centre (MUMC), Maastricht, The Netherlands

**Keywords:** Liver, Randomized controlled trials

## Abstract

The objective of this randomized controlled trial (RCT) was to assess the impact of rifaximin on the course of liver function, liver regeneration and volumetric recovery in patients undergoing major hepatectomy. The ARROW trial was an investigator initiated, single-center, open-label, phase 3 RCT with two parallel treatment groups, conducted at our hepatobiliary center from 03/2016 to 07/2020. Patients undergoing major hepatectomy were eligible and randomly assigned 1:1 to receive oral rifaximin (550 mg twice daily for 7–10 or 14–21 days in case of portal vein embolization preoperatively and 7 days postoperatively) versus no intervention. Primary endpoint was the relative increase in postoperative liver function measured by LiMAx from postoperative day (POD) 4 to 7. Secondary endpoint were the course of liver function and liver volume during the study period as well as postoperative morbidity and mortality. Between 2016 and 2020, 45 patients were randomized and 35 patients (16 individuals in the rifaximin and 19 individuals in the control group) were eligible for per-protocol analysis. The study was prematurely terminated following interim analysis, due to the unlikelihood of reaching a significant primary endpoint. The median relative increase in liver function from POD 4 to POD 7 was 27% in the rifaximin group and 41% in the control group (*p* = 0.399). Further, no significant difference was found in terms of any other endpoints of functional liver- and volume regeneration or perioperative surgical complications following the application of rifaximin versus no intervention. Perioperative application of rifaximin has no effect on functional or volumetric regeneration after major hepatectomy (NCT02555293; EudraCT 2013-004644-28).

## Introduction

Liver resection (LR) is a major cornerstone in the therapy of primary and secondary liver tumors, displaying compelling long-term oncological outcomes in comparison to interventional or medical treatment in various hepatobiliary and oncological diseases^1–4^. Despite its broad acceptance, LR remains a highly invasive procedure with reported mortality rates up to 15% depending on patient selection, indication and the particular technical procedure^5,6^. Especially major LR—defined by the surgical removal of more than 2 liver segments—is associated with significant postoperative morbidity and mortality due to postoperative liver failure (POLF)^7,8^. POLF is considered to be an acquired deterioration in the ability of the liver to maintain its synthetic, excretory and detoxifying function after LR^9^. POLF is further reported to be the main driver of postoperative morbidity and mortality in these patients and occurs in up to 10% of patients undergoing major LR. Subsequently, improving perioperative liver function and enhancing liver regeneration after LR has been a research focus of the last decades^10^.

Liver regeneration is regulated by a complex interaction of hepatocytes and non-parenchymal cells directed by cytokines, growth hormones and metabolic factors^11^. Over the last years, the bidirectional relationship between the liver and the intestine, the so-called gut-liver axis, and its role in liver regeneration and disease are gaining more and more attention. LR is known to affect the integrity of the gut epithelial barrier, facilitate the translocation of bacteria and bacterial products to the liver were these products trigger an inflammatory response^12^. As liver regeneration is strongly inhibited by hepatic inflammation, any medical interventions to reduce inflammation in the early postoperative course appear reasonable^13,14^.

Rifaximin is a rifamycin derivative with a broad therapeutic range and approved for the treatment of gastrointestinal infections^15^. Further, rifaximin has shown efficiency to maintain remission from hepatic encephalopathy and reduce the risk of hospitalization involving hepatic encephalopathy^16^. Thus, considering the aforementioned direct link between gut microbiota translocation to the liver as a pathophysiological event following major LR and its potential adverse role in substantial liver dysfunction and POLF, we hypothesized that perioperative antibiotic treatment with rifaximin may improve postoperative liver function and reduce morbidity after major LR.

Due to the low bioavailability of rifaximin with less than 0.5% of the oral dose being intestinally absorbed, there is a low risk of systemic toxicity, allowing a safe use in the perioperative setting^15^. In this RCT, we systematically assessed the impact of rifaximin on the course of liver function, liver regeneration and volumetric recovery in patients undergoing major LR. The RCT is presented in accordance with the Consolidated Standards of Reporting Trials (CONSORT) guidelines.

## Material and methods

### Study design

ARROW (Administration of Rifaximin to improve Liver Regeneration and Outcome following Major Liver Resection) is a randomized, controlled, single-center, open-label superiority phase 3 trial with 2 parallel treatment groups. The study is an investigator-initiated trial conducted according to the requirements of the German Medicinal Products Act (Arzneimittelgesetz-AMG). The study protocol was approved by the German Federal Institute for Drugs and Medical Devices and was registered with ClinicalTrials.gov and EudraCT (NCT02555293, first registration 21/09/2015; EudraCT 2013-004644-28, first registration 18/03/2014). The RWTH Aachen University acted as the responsible sponsor for the trial. The ARROW trial was approved by the institutional review board of the RWTH Aachen (EK 13-129) and all necessary regulatory approvals were obtained. There were no major protocol amendments during the study period impacting trial design or trial objectives. Informed consent was obtained from every patient and the trial has been conducted in accordance with the current version of the Declaration of Helsinki, and the good clinical practice guidelines (ICH-GCP). The ARROW trial was conducted and reported according to the CONSORT guidelines.

### Study participation

Patients aged between 18 and 80 years who were scheduled for major LR were eligible if they additionally presented with an BMI between 18 and 40 and were assessed with a performance status I to III according to the American society of anesthesiologist (ASA) classification. Patients with the requirement of concomitant extrahepatic surgical procedures, hyperthermic intraperitoneal chemotherapy (HIPEC) or associating liver partition and portal vein ligation for staged hepatectomy (ALPPS) were ineligible for trial participation.

### Randomization and masking

Patients were randomly assigned 1:1 to the rifaximin group or the control group. Treatment was not masked and no placebo was used in the control group. Random allocation was carried out by a computer algorithm (Study Management Tool, RWTH Aachen University, Germany) that stratified participants by the preoperative requirement of portal vein embolization.

### Procedures

**Figure 1:**
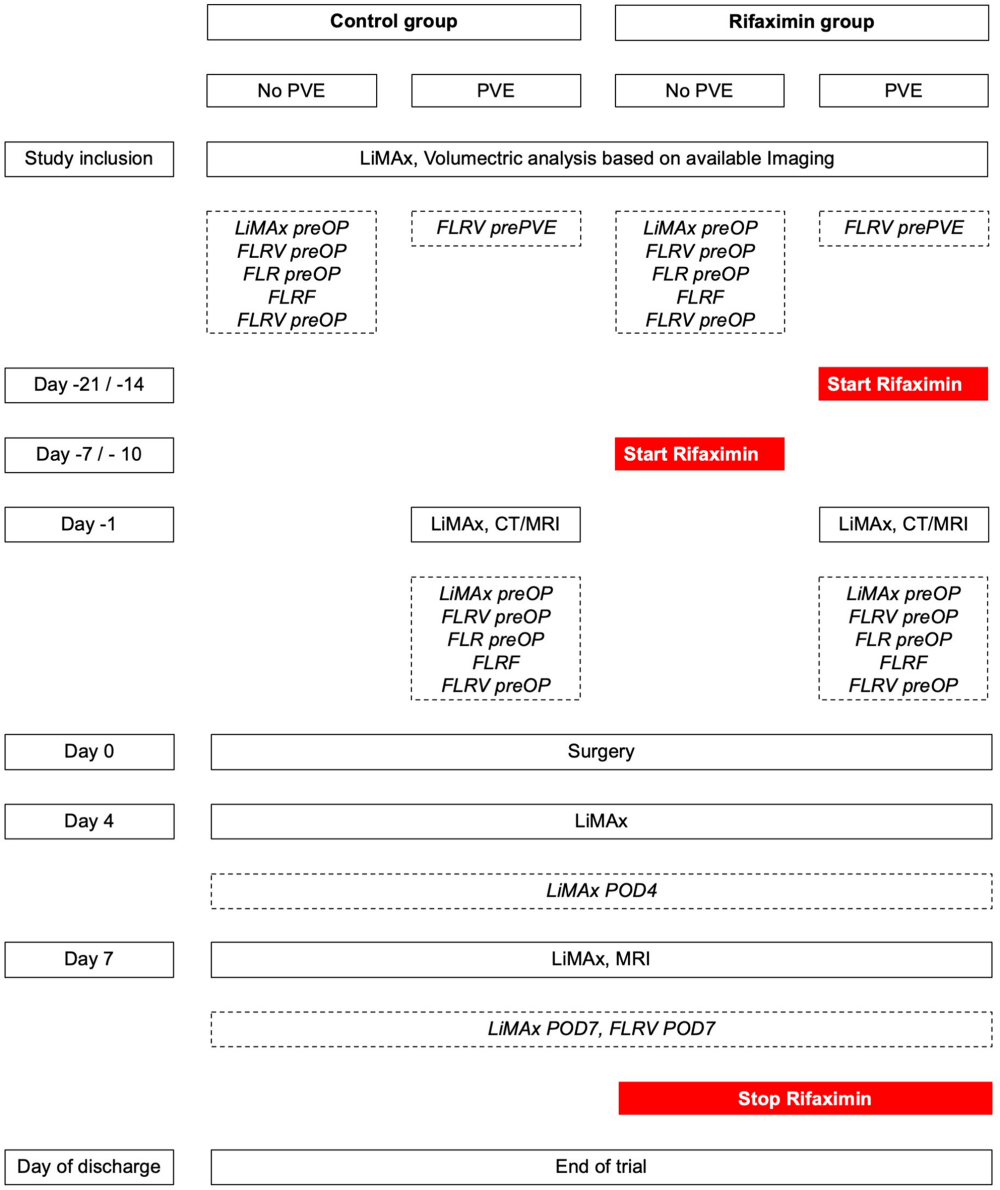
Trial overview. A reduced overview of the trial and all included study visits. Study events and tests are depicted in continues rectangles and measured variables with importance for statistical analysis in dashed rectangles. FLRF, future liver remnant function; FLRV, future liver remnant volume; LiMAx, maximum liver function capacity; MRI, Magnetic resonance imaging; POD, postoperative day; prePVE, prior to PVE; preOP, prior to surgery; PVE, portal vein embolization.

All patients were screened and recruited in the local outpatient department. Liver function according to LiMAx (maximum liver capacity) was determined and computed-tomography (CT) volumetry of the future liver remnant (FLR) was carried out. In need of preoperative hypertrophy induction, portal vein embolization (PVE) was scheduled. In these particular patients, LiMAx and volumetric assessment were repeated one day prior to actual LR. In the treatment group, rifaximin (550 mg) was given twice a day for 14 to 21 days in case of PVE or 7 to 10 days in cases without preoperative PVE prior to LR, respectively. After LR, rifaximin was continued until the postoperative day (POD) 7 and then discontinued. Patients participating in the trial were regularly visited until discharge and underwent LiMAx on the POD 4 and 7 as well as a magnetic resonance imaging (MRI) on POD 7. A reduced trial overview including all study visits is shown in Fig. 1.

### Methods

Liver function was determined by the LiMAx which represents a dynamic C13-breath test reflecting enzymatic liver function capacity. During the test, a bodyweight-adjusted intravenous ^13^C-labeled methacetin bolus injection and continuous measurement of the ^13^CO_2_/^12^CO_2_ concentration ratio using a special device (FLIP, Humedics GmbH, Berlin, Germany) is performed as previously described^17^. LiMAx values > 315 μg/kg/h are considered normal^18^.

CT- or MRI-based volumetry was carried out using a dedicated software (IntelliSpace Portal 8.0 software, Philips healthcare, Amsterdam, The Netherlands). After manual delineation of margins in every slide, total liver volume (TLV), tumor volume (TV) and future liver remnant volume (FLRV) were subsequently computed automatically. TV was considered as non-functional liver parenchyma for all functional calculations. Finally, the FLR was computed by the following formula:

$$ FLR \left[ {{\% }} \right] = \frac{FLRV}{{TLV - TV}} \times 100 $$

Direct postoperative liver function (future liver remnant function, FLRF) is estimated on the basis of preoperative LiMAx values and the results of the volumetric liver analysis using the following formula as previously described^8^:

$$ FLRF \left[ {{\mu g}/{\text{kg}}/{\text{h}}} \right] = FLR \times LiMAx $$

PVE was carried out using a percutaneous transhepatic ipsilateral approach as previously described^19^. Briefly, a catheter was inserted into the right portal vein by transhepatic CT-guided puncture of the right portal branch. Embolization of the right portal branches was carried out with a mixture of n-butyl-cyanoacrylate (Braun, Tuttlingen, Germany) and lipiodol (Guerbet, Roissy, France) in a ratio of 1:2 to 1:3. Successful embolization was confirmed through repeated portography.

LR was carried in accordance to clinical standards as previously described^20^.

An intraoperative ultrasound was performed to visualize the local tumor spread and other suspicious lesions. Parenchymal transection was carried out using the Cavitron Ultrasonic Surgical Aspirator (CUSA, Integra LifeSciences, Plainsboro NJ, USA) with low central venous pressure (CVP) and intermittent Pringle maneuvers if necessary. In laparoscopic hepatectomy, parenchymal transection was commonly performed by Thunderbeat (Olympus K.K., Tokyo, Japan), Harmonic Ace (Ethicon Inc. Somerville, NJ, USA) or laparoscopic CUSA (Integra life sciences, New Jersey, USA) in combination with vascular staplers (Echelon, Ethicon, Somerville, New Jersey, USA) or polymer clips (Teleflex Inc., Pennsylvania, USA). The anesthesiologic management was based on a restrictive fluid intervention strategy ensuring a low central venous pressure (CVP) during parenchymal dissection.

### Outcomes

The primary outcome of the trial was functional recovery after major LR, defined as the percental increase of LiMAx measured on POD 7 in relation to LiMAx measured on POD 4. Secondary outcomes were volumetric recovery, defined as percental increase of FLRV measured on POD 7 in relation to preoperatively determined FLRV, the hypertrophy of the FLRV after PVE and course of liver function over time determined by LiMAx as well as postoperative morbidity and mortality.

### Statistical analysis

An a priori sample size calculation of the trial was based on an estimated change of 30% of LiMAx values in the treatment group compared to the control and a dropout rate of 10% based on the findings of Rayes et al. investigating liver regeneration after right hepatectomy determined by LiMAx in the context of perioperative administration of probiotics^21^. As such, 96 patients were required to detect a statistically significant difference between the groups with a two-sided significance level of 5% and 0.90 power. Extensive statistical group comparisons were conducted between the rifaximin and control group. Categorical data are presented as numbers and percentages and are statistically analyzed using the chi-squared test, fisher’s exact test or linear-by-linear association in accordance to scale and number of cases. Continuous variables are presented as median and interquartile range and compared by the Mann–Whitney-U-test. Perioperative complications were classified according to the Clavien-Dindo scale^22^. The level of significance was set to *p* < 0.05 and *p*-values are given for two-sided testing. Analyses were carried out using SPSS Statistics 24 (IBM Corp., Armonk, NY, USA).

### Early termination

In 08/2020, an interims analysis was conducted to ensure trial safety. Here, no relevant difference was found regarding the primary outcome of the trial (LiMAx increase from POD 4 to POD 7) by the medical advisor board. Subsequently, the trial was prematurely stopped and the incomplete dataset was analyzed.

### Role of the funding source

ARROW was an investigator-initiated trial predominantly using internal departmental funds. However, limited external funding covering the study medication was provided by Norgine GmbH (Wettenberg, Germany). Norgine GmbH had no role in running of the study, data collection, analysis and interpretation or writing of the publication. Upon completion of all trial data, JB, TL and UPN had full access to all the data and the corresponding authors had final responsibility for the decision to submit for publication.

## Results

**Figure 2:**
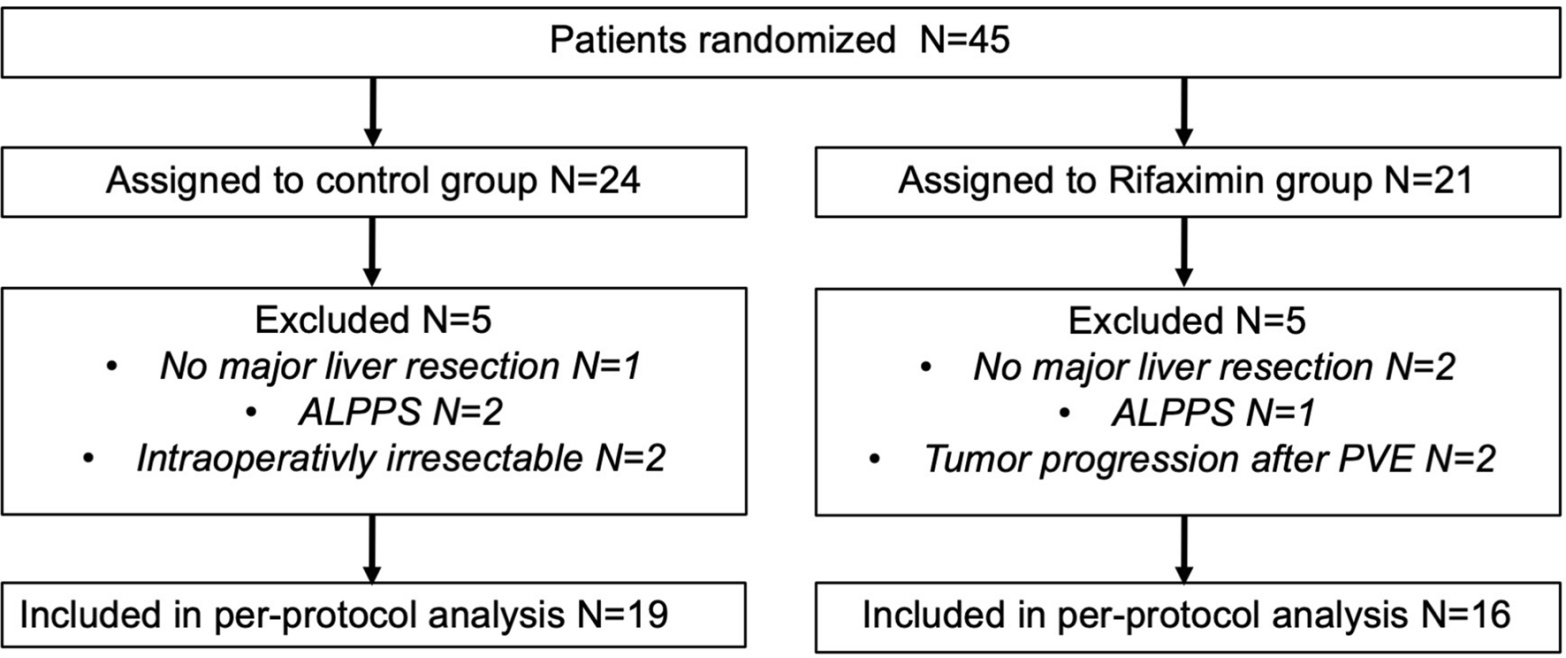
Trial profile. ALPPS, Associating liver partition with portal vein ligation for staged hepatectomy; PVE, portal vein embolization.

Between 03/2016 and 07/2020, at total of 45 patients were enrolled in the trial and randomly assigned to the control (n = 24) and rifaximin group (n = 21). Of all randomized patients, a set of 10 individuals were excluded from the trial as they were finally not treated by major LR (n = 3), underwent ALPPS (n = 3), were intraoperatively assessed as technically not resectable (n = 2) or showed tumor progression after PVE which precluded further surgical therapy (n = 2). No further withdrawal from trial treatment or consent were recorded during the study period. All patients underwent the trial as scheduled and study medication was completed in all individuals of the rifaximin group. As such, 19 patients in the control and 16 patients in the rifaximin group were eligible for a per-protocol analysis. A detailed trial profile is shown in Fig. 2.

### Patients’ characteristics

The groups were well balanced regarding clinical characteristics with no observed difference in gender (*p* = 0.830), age (*p* = 0.481), BMI (*p* = 0.301) and ASA categorization (*p* = 0.501). While there was a higher rate of perihilar cholangiocarcinoma (pCCA) and a lower rate of colorectal liver metastases in the control group (CRLM; 7/19 vs. 2/16 and 4/19 vs. 9/16 respectively), this tendency did not gain statistical significance (*p* = 0.131). Also, no difference was observed regarding laboratory liver function and clinical chemistry (Table 1). Further, the applied surgical procedures (*p* = 0.613) as well as operative morbidity and mortality (*p* = 0.731) were comparable between the groups. The total number of AEs and SAEs were 33 and 16 in the control and 29 and 14 in the rifaximin group (*p* = 0.688; *p* = 0.284). A detailed overview of patients’ characteristics is given in Table 1.

**Table 1:** Comparative analysis of the trial cohort.

Variables	Control group vs. rifaximin group analysis		
	Control group (n = 19)	Rifaximin group (n = 16)	*p*
**Demographics**			
Sex, m/f (%)	10 (52.6)/9 (47.4)	9 (56.3)/7 (43.8)	.830
Age (years)	60 (55–68)	62 (56–74)	.481
BMI (kg/m^2^)	27 (24–31)	25 (24–28)	.301
ASA, n (%)			.515
I	1 (5.3)	0	
II	10 (52.6)	8 (50.0)	
III	8 (42.1)	8 (50.0)	
Diagnosis, n (%)			.131
Perihilar Cholangiocarcinoma	7 (36.8)	2 (12.5)	
Intrahepatic Cholangiocarcinoma	1 (5.3)	3 (18.8)	
Hepatocellular Carcinoma	3 (15.8)	1 (6.3)	
Colorectal Liver Metastases	4 (21.1)	9 (56.3)	
Adenoma	1 (5.3)	1 (6.3)	
Hemangioma	3 (15.8)	0	
Preoperative chemotherapy, n (%)	3 (15.8)	4 (25.0)	.497
Preoperative PVE, n (%)	8 (42.1)	8 (50.0)	.640
**Laboratory liver function and clinical chemistry**			
AST (U/l)	35 (26–48)	29 (26–44)	.443
ALT (U/l)	45 (23–63)	29 (18–46)	.271
GGT (U/l)	201 (77–331)	111 (46–504)	.317
Total bilirubin (mg/dl)	0.49 (0.34–0.92)	0.41 (0.35–0.59)	.441
Platelet count (/nl)	310 (246–391)	288 (211–327)	.151
Alkaline Phosphatase (U/l)	161 (95–262)	140 (94–321)	.683
Prothrombin time (%)	99 (86–107)	97 (91–108)	.935
Hemoglobin (g/dl)	13.1 (11.7–14.6)	12.7 (12.1–13.9)	.502
Creatinine (mg/dl)	0.8 (0.7–1.0)	0.8 (0.7–1.0)	.883
**Operative characteristics**			
Operative procedure, n (%)			.613
Right hepatectomy	9 (47.4)	7 (43.8)	
Left hepatectomy	3 (15.8)	1 (6.3)	
Extended right hepatectomy	4 (21.1)	5 (31.3)	
Extended left hepatectomy	0	1 (6.3)	
Right trisectionectomy	2 (10.5)	1 (6.3)	
Left trisectionectomy	1 (5.3)	0	
Central resection	0	1 (6.3)	
Operative time (minutes)	375 (283–453)	386 (279–437)	.935
Intraoperative blood transfusion, n (%)	8 (42.1)	4 (25.0)	.288
Intraoperative FFP, n (%)	10 (52.6)	5 (31.3)	.203
Postoperative complications, n (%)			.731
No complications	7 (36.8)	6 (37.5)	
I	1 (5.3)	4 (25.0)	
II	5 (26.3)	2 (12.5)	
IIIa	2 (10.5)	1 (6.3)	
IIIb	1 (5.3)	1 (6.3)	
IVa	0	0	
IVb	1 (5.3)	1 (6.3)	
V	2 (10.5)	1 (6.3)	
ISGLS liver failure			.561
None	14 (73.7)	12 (75.0)	
Grade A	4 (21.1)	3 (18.8)	
Grade B	1 (5.4)	0	
Grade C	0	1 (6.3)	
Number of AE, total per trial arm	35	30	.875
Number of SAE, total per trial arm	17	15	.241
**Dynamic liver function analysis**			
LiMAx preOP (µg/kg/h)	378 (280–588)	461 (325–543)	.567
FLRF (µg/kg/h)	137 (104–229)	178 (155–226)	.125
LiMAx POD4 (µg/kg/h)	146 (72–264)	175 (135–300)	.142
LiMAx POD7 (µg/kg/h)	214 (107–287)	244 (176–373)	.483
Increase FLRF to POD4 (%)	− 6 (− 5–40)	13 (0–56)	.331
Increase FLRF to POD7 (%)	24 (9–94)	54 (3–89)	.815
Increase POD4 to POD7 (%)	41 (3–99)	27 (− 3–63)	.399
**Volumetric analysis**			
FLRV prePVE (ml)*	543 (403–782)	398 (381–566)	.234
FLRV preOP (ml)*	662 (532–769)	558 (474–735)	.279
Increase prePVE to preOP (%)*	32 (5–36)	33 (18–44)	.574
FLRV preOP (ml)	637 (503–798)	676 (509–939)	.987
FLR preOP (%)	39 (33–44)	41 (34–54)	.333
FLRV POD7 (ml)	962 (831–1352)	827 (711–982)	.140
Increase FLRV preOP to FLRV POD7	52 (27–72)	45 (17–50)	.180
**Cytokine data**			
TNFa (ng/l)			
Visit 1 (study inclusion)	6.3 (4.5–8.2)	5.1 (4.5–10.4)	.825
Visit 3 (preOP)	7.2 (5.6–9.1)	7.1 (5.6–11.3)	.798
Visit 4 (POD4)	7.3 (6.4–8.8)	7.2 (5.3–12.4)	.953
Visit 5 (POD7)	7.2 (5.7–9.0)	6.7 (4.9–9.1)	.597
IL-6 (pg/ml)			
Visit 1 (study inclusion)	6.1 (2.6–9.2)	7.1 (3.4–9.7)	.746
Visit 3 (preOP)	7.0 (1.5–7.9)	7.6 (1.9–11.0)	.635
Visit 4 (POD4)	36.6 (23.9–63.3)	34.3 (18.1–59.5)	.468
Visit 5 (POD7)	43.3 (30.0–84.3)	29.4 (17.8–50.7)	.144

Data presented as median and interquartile range if not noted otherwise. Categorical data were compared using the chi-squared test, fisher’s exact test or linear-by-linear association according to scale and number of cases. Data derived from continuous variables of different groups were compared by Mann–Whitney-U-Test.

*Data only shown for patients who underwent PVE. AE, adverse event; ALT, alanine aminotransferase; AP, alkaline phosphatase ASA, American society of anesthesiologists classification; AST, aspartate aminotransferase; BMI, body mass index; CRP, c-reactive protein; FFP, fresh frozen plasma; FLR, future liver remant; FLRF, future liver remnant function; FLRV, future liver remant volume; GGT, gamma glutamyltransferase; IL, Interleukin; ISGLS, International Study Group for Liver Surgery; LiMAx, Liver function capacity; POD, postoperative day; preOP, preoperative day 1; PVE, portal vein embolization; SAE, serious adverse event; TNF, tumor necrosis factor.

### Course of liver function

**Figure 3:**
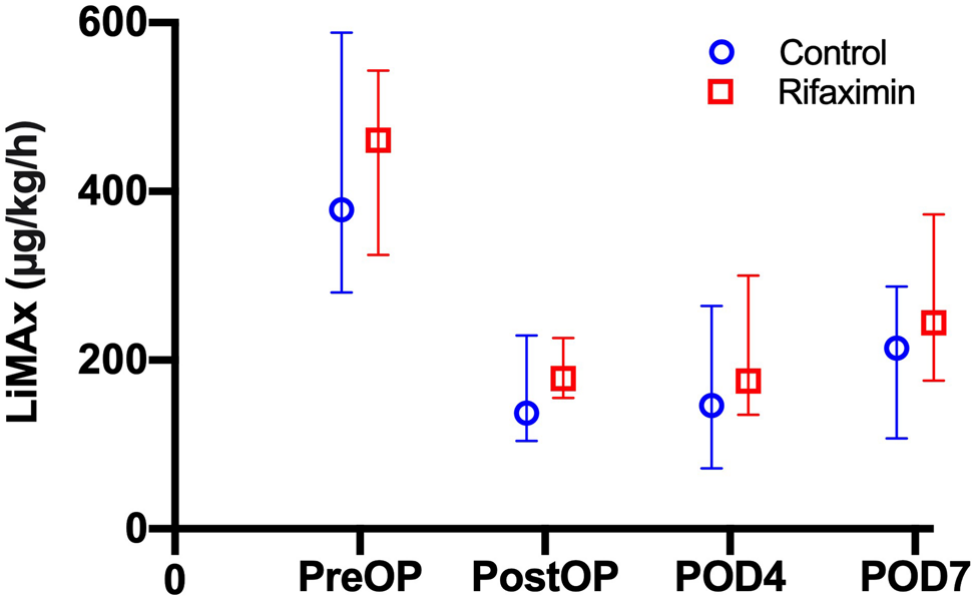
Course of liver function with respect to trial group. Liver function assessed with LiMAx during the trial period is presented as median and interquartile range. Liver function was significantly reduced due to liver resection and did subsequently recover in the postoperative period. However, no time point showed statistical significance between the Rifaximin and control group (preOP: *p* = 0.567; postOP: *p* = 0.125; POD 4: *p* = 0.142; POD 7: *p* = 0.483). LiMAx, maximum liver function capacity; POD, postoperative day; postOP, after surgery; preOP, prior to surgery.

The median preoperative LiMAx was 378 µg/kg/h in the control and 461 µg/kg/h in the Rifaximin group (*p* = 0.567). The median estimated FLRF based on preoperative LiMAx and volumetry of the FLR was calculated to be 137 µg/kg/h in the control and 178 µg/kg/h in the rifaximin group (*p* = 0.125). In the postoperative setting, median LiMAx was 146 µg/kg/h on POD 4 and 214 µg/kg/h on POD 7 in the control as well as 175 µg/kg/h and 244 µg/kg/h in the rifaximin group (*p* = 0.142; *p* = 0.483). In the control group the median increase from FLRF to POD 4 was -6% and 24% to POD 7, while in the increase from FLRF to POD 4 was 13% and 54% to POD 7 in the rifaximin group (*p* = 0.331; *p* = 0.815). The relative increase of liver function from POD 4 to POD 7 was 41% in the control and 27% in the rifaximin group (*p* = 0.399). More details are presented in Table 1 and Fig. 3.

### Course of liver volume

**Figure 4:**
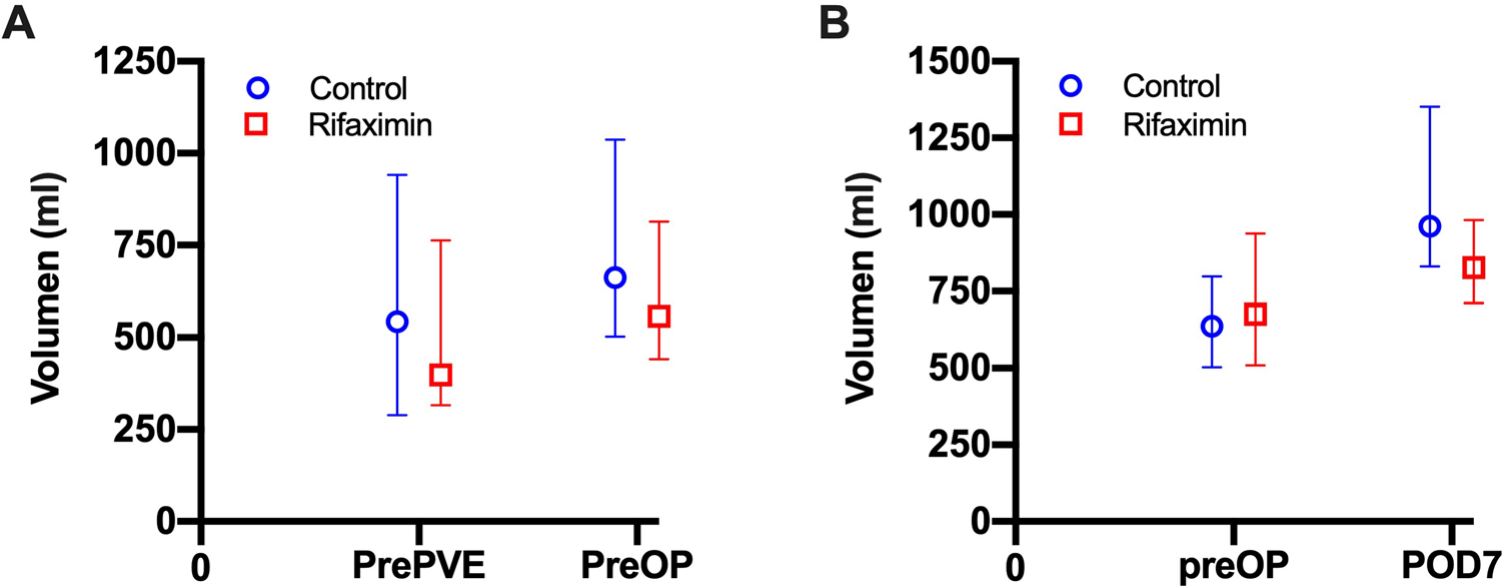
Course of future liver remnant liver volume with respect to trial group. FRLV was assessed on different time points during the trial period is presented as median and interquartile range (**A**) Volumetric growth after PVE. FRLV subsequently increased after PVE in both groups with no statistical in any time point (prePVE: *p* = 0.234; preOP: *p* = 0.279; only patients undergoing PVE were analyzed). (**B**) Volumetric growth after liver resection. FRLV increased after surgery in both groups with no statistical in any time point (postOP: *p* = 0.987; POD 7: *p* = 0.140). FLRV, future liver remnant volume; POD, postoperative day; prePVE, prior to PVE; preOP, prior to surgery.

The median estimated FLRV was 637 ml in the control and 676 ml in the rifaximin group (*p* = 987). On POD 7, the median measured FRLV was 962 ml in the control as well as 827 ml in the rifaximin group (*p* = 0.140) which translates to a median postoperative increase from FLRV to POD 7 of 52% in the control and 45% in the rifaximin group (*p* = 0.180). A similar sub-group analysis was carried out for PVE patients (n = 16, 8/8) exclusively investigating hypertrophy after PVE. Here, the median volume increase of the FRLV was 32% in the control and 33% in the rifaximin group (*p* = 0.574). More details are presented in Table 1 and Fig. 4.

### Cytokine release

**Figure 5:**
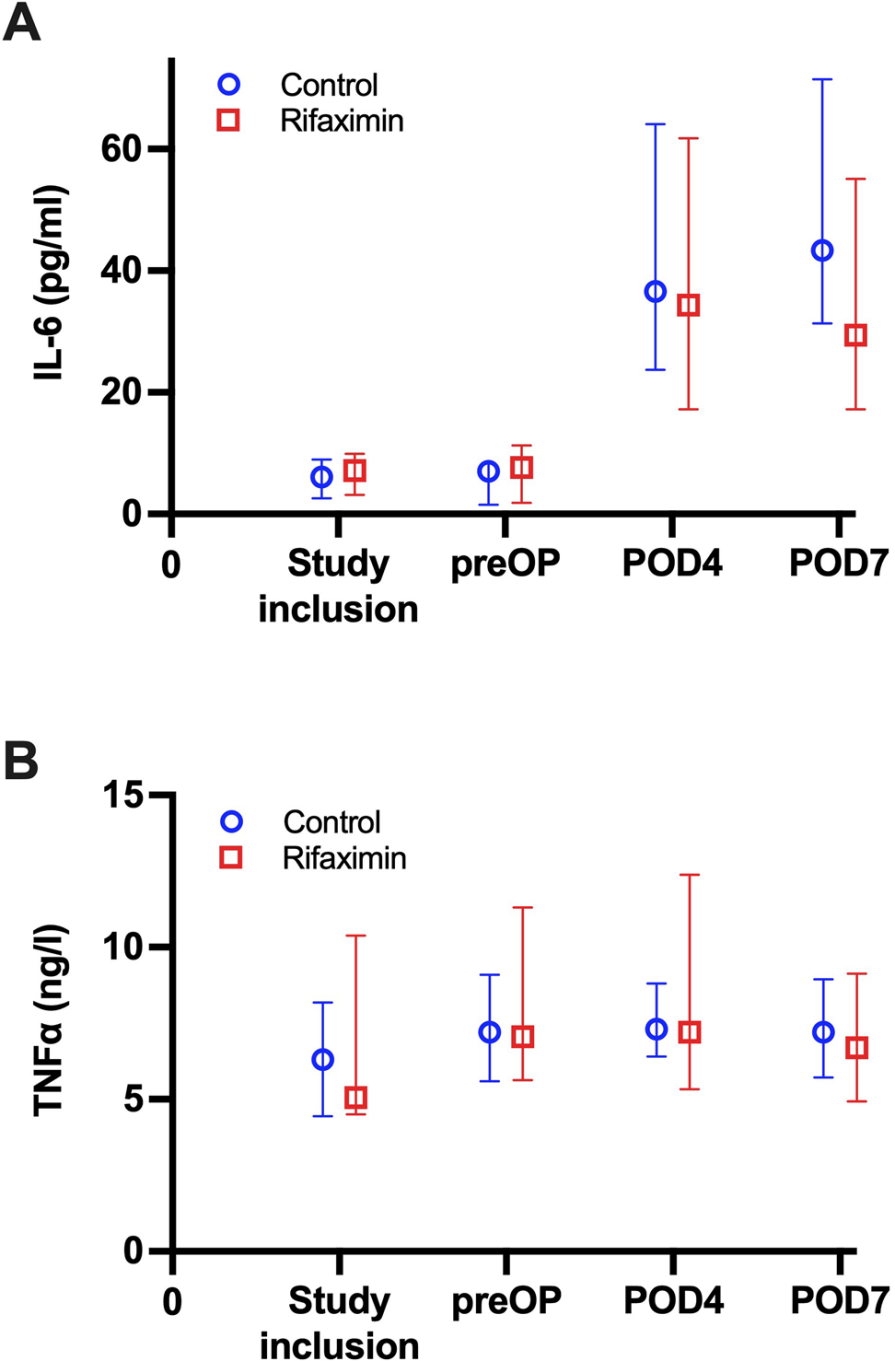
Course of cytokines with respect to trial group. (**A**) IL-6. The course of serum IL-6 levels is presented as median and interquartile range. There was no significant difference in IL-6 levels between the Rifaximin and control group at any of the time points (study inclusion: *p* = 0.825; preOP: *p* = 0.798; POD 4: *p* = 0.953; POD 7: *p* = 0.597). (**B**) TNFα. The course of TNFα is presented as median and interquartile range. Also, no time point showed statistically significant difference between the Rifaximin and control group (study inclusion: *p* = 0.746; preOP: *p* = 0.635; POD 4: *p* = 0.468; POD 7: *p* = 0.144). IL, Interleukin; POD, postoperative day; preOP, prior to surgery. TNF, tumor necrosis factor.

To explore to underlying effects of rifaximin, tumor necrosis factor alpha (TNFα) and interleukin 6 (IL-6) were assessed at pre-defined time points over the course of the trial. Here, no significant between-group differences were observed for IL-6 and TNFα at any time point. A detailed overview of the course of serum cytokine release is provided in Table 1 and Fig. 5.

## Discussion

In the ARROW trial, we investigated the impact of the perioperative application of rifaximin on functional and volumetric recovery after LR. As no effect of the study medication on the primary readout of the trial—liver function increase from POD 4 to POD 7 measured by LiMAx—was observed in the interim analysis, the trial was prematurely discontinued and completely analyzed using the available data. Based on the results of the trial, we were not able to demonstrate a significant benefit in functional regeneration or volumetric increase as well as in perioperative morbidity and mortality in this dataset.

The gut-liver axis, which refers to the bidirectional relationship between the intestinal microbiome, the gut and the liver, has been in the focus of gastrointestinal research in the last decade^12^. The microbiome is the first interface between environment and the gut barrier and has been shown to be influenced by dietary habits, ethanol and certain drugs (i.e. antibiotics among others)^23–25^. Treatment with antibiotics can significantly alter the intestinal microbiome and can therefore play a role in liver damage following surgical resection and liver transplantation. For example, administration of polymyxin B is associated with the reduction of total parenteral nutrition induced steatosis in both rats and humans^26^. The pathophysiological background of this observation might be explained by bacterial translocation which is the passage of bacteria or bacterial products to the liver via the portal circulation^27^. Here, bacterial components can increase the expression of specific receptors, e.g. Toll-Like Receptors (TLRs). TLRs can bind pathogen‐associated molecular patterns (PAMPs) and damage-associated molecular patterns (DAMPs) leading to increased gene expression of pro-inflammatory mediators such as TNFα, IL-1β, and interferons^28^. These effects are further facilitated by an increased gut permeability which is a well-known pathophysiological mechanism in a broad spectrum of liver diseases^29^.

The non-absorbable rifaximin is a potent treatment in various gastrointestinal diseases and has beneficial clinical effects in irritable bowel syndrome, treatment and prevention of traveler’s diarrhea, small intestinal bacterial overgrowth, hepatic encephalopathy and diverticular disease^30^. The underlying physiological mechanisms are still a matter of debate. Rifaximin appears to have a minimal negative impact on overall gut microbiota, only leading to a transient change in the concentrations of GI bacteria during therapy^31^. Further, studies on rifaximin in cell lines indicate an ability to alter cytokine expression profiles (e.g. IL-8, etc.) suggesting an anti-inflammatory activity^32^. Also reduced pathogen adherence in cell line studies investigating epithelial cell physiology has been attributed to rifaximin exposure^33^. Additionally, rifaximin prevented the elevation of IL-17, IL-6, TNFα and the stress-induced increase in mucosal inflammation and gut permeability^31,34^. While TNFα and IL-6 themself can increase gut permeability by affecting tight junctions allowing bacterial translocation, both cytokines also play a major role in liver homeostasis^35^. In the context of acute liver failure, high levels of circulating IL-6 and TNFα are associated with impaired outcome in some studies, while in the hepatic compartment itself IL-6 seems to be protective as it downregulates TNFα-induced hepatic apoptosis^36,37^. Also, both TNFα and IL-6 are known as pivotal regulators during the early phase of liver regeneration as they modulate the interaction between non-parenchymal cells and hepatocytes inducing the priming process, i.e., the G0/G1 transition of hepatocytes and production of hepatic growth factor (HGF)^38,39^. As no differences in the release characteristics of TNFα and IL-6 were observed between treatment and control group in our study, we were not able to further elucidate the effects of rifaximin on cytokines in the scenario of our clinical RCT. While the physiological basis of its effect remains to be fully unraveled, rifaximin appears to interfere with the gut-liver axis by modifying the microbiome, enhancing the epithelial gut barrier and modulating inflammatory response^34^. This observation is further supported by its various clinical applications^30^.

However, within the setting of this RCT, investigating rifaximin in the context of major LR, we were not able to detect any notable effect on liver function or volumetric recovery. Reasons for this negative observation might be attributed to the trial design and the clinical context of major LR. As liver dysfunction or POLF usually arises after significant loss of the liver parenchyma, we decided to restrict the trial to individuals undergoing major LR^40^. Despite careful preoperative patient selection, major LR remains an invasive procedure with significant perioperative morbidity as illustrated in our cohort by the rate of major morbidity (defined as any complications rated > Clavien Dindo II) in 32% (6/19) in the control and 25% (4/16) in the rifaximin group. Perioperative complications are known to have a strong effect on liver function and regeneration^41^. LiMAx is a precise diagnostic tool to measure liver function and has proven its diagnostic and predictive abilities in various clinical scenarios^8,17,42–44^. This is also shown in our trial with both groups displaying a significant drop in liver function due to LR and a continuous recovery in the postoperative course (Fig. 2). LiMAx is further capable to measure liver regeneration after LR and is able to detect impaired regeneration in case of biliary leakage, a common septic complication after LR^45^. It is therefore plausible and also provides a partial explanation for our findings that surgical complications often have a much stronger detrimental effect on the functional regeneration than the assumed positive effect achieved by the perioperative application of rifaximin. Of note, a comparable effect was observed in a study of Rayes et al. investigating probiotics^21^. In this RCT, patients scheduled for right hepatectomy received enteral nutrition with fibers only or fibers supplemented by probiotics starting the day before surgery and continuing until the 10th POD. Primary study endpoint was, likewise in our trial, the increase of liver function determined by LiMAx. Here, a slightly improved liver regeneration in the treatment group was observed and in a sub-group of patients excluding perioperative complications the positive effect in the treatment group was even more obvious. These observations further underline the fundamental role of perioperative morbidity in liver regeneration. This would also implicate that the outcome of any clinical trial evaluating rifaximin’s ability in improving postoperative liver regeneration may be influenced by minor or major postoperative complications and, therefore postoperative liver function characteristics as trial endpoint should always be interpreted in the context of perioperative morbidity.

Our trial did also not observe any difference in volumetric recovery after surgery which does underline the negligible or no potential effect of rifaximin on volume regeneration after LR. Interestingly, we were further not able to demonstrate an impact on hypertrophy of the FLR after PVE in a small subgroup analysis of patients requiring PVE in the preoperative setting. This is in particular interesting as this small sample set (n = 16) experienced no major complications between PVE and LR which would interfere with a potentially benefit of rifaximin treatment. One might argue that given the notable risk for complications after major LR, patients undergoing minor LR might be more suitable to assess the potential impact of rifaximin. However, liver function is usually less altered after minor LR and the likelihood of significant dysfunction or POLF is considerably less making a medical intervention to boost liver function postoperatively clinically not relevant.

To date rifaximin shows major therapeutic effects in the prevention of hepatic encephalopathy or the long-term treatment of small intestinal bacterial overgrowth. However, there is currently no evidence supporting a therapeutic role in acute or acute-on-chronic liver failure which might be a comparable clinical situation to LR^30^. It is therefore assumable that the significant clinical impact of rifaximin in various gastrointestinal disease is rather attributed to a prolonged modulation of the liver-gut axis than to a rapid improvement in liver function. Based on this, it is possible that a longer application of rifaximin would result in a more meaningful effect. However, the treatment duration was already 7 to 10 days prior to the operation (14 to 21 days in case of PVE) accompanied by 7 days after the LR in both, patients with and without PVE. An even longer preoperative application might not be practicable in an oncological setting as it might delay surgery and risk tumor related complications in these patients (e.g. local tumor progression or metastases).

Despite showing no significant effect in our RCT, the use of rifaximin was safe with no relevant side effects. SAE and AE were equally distributed between the groups and no association between SAE and AE and potential side effects of rifaximin were observed by the medical advisor board indicating a safe usage in the perioperative setting.

Our trial has certainly some obvious limitations which have to be discussed critically. First, the trial had to be terminated prematurely which resulted in a smaller data set, leaving several questions unanswered. Additionally, we have to report a high dropout rate reflecting the clinical reality in oncological liver surgery. Second, the trial was conducted in a single center based on the authors’ distinct clinical management in LR. Third, some parameters were calculated and not measured (FLRF and FLRV). However, these calculations are commonly conducted in liver surgery and have shown their accuracy and predictive ability in previous reports^8,46^. Also, the primary endpoint of the study which was defined as the functional increase from POD 4 to POD 7 was solely based on measured variables.

The ARROW trial is the first clinical trial to investigate the effect of perioperative administration of rifaximin on liver regeneration after LR. Considering the aforementioned limitations, we conclude that perioperative application of rifaximin is safe, but does not improve functional or volumetric regeneration after major LR.

## Data Availability

Available upon request. JB and UPN had full access to the data and act both as guarantor for the data.

## Abbreviations

AE: Adverse event
ALPPS: Associating liver partition and portal vein ligation for staged hepatectomy
ALT: Alanine aminotransferase
AP: Alkaline phosphatase
ASA: American society of anesthesiologists
AST: Aspartate aminotransferase
BMI: Body mass index
CONSORT: Consolidated standards of reporting trials
CRLM: Colorectal liver metastases
CT: Computed tomography
CVP: Central venous pressure
DAMP: Damage-associated molecular patterns
FFP: Fresh frozen plasma
FLR: Future liver remnant
FLRF: Future liver remnant function
FLRV: Future liver remnant volume
GGT: Gamma glutamyl transferase
IIT: Investigator initiated trial
IL: Interleukin
LiMAx: Maximum liver function capacity
MRI: Magnetic resonance imaging
NAFLD: Nonalcoholic fatty liver disease
NASH: Nonalcoholic steatohepatitis
pCCA: Perihilar cholangiocarcinoma
POD: Postoperative day
prePVE: Prior to PVE
preOP: Prior to surgery
PAMP: Pathogen‐associated molecular patterns
PVE: Portal vein embolization
RCT: Randomized controlled trial
SAE: Serious adverse event
TLR: Toll-like receptor
TNF: Tumor necrosis factor
RWTH: Rheinisch-Westfälische Technische Hochschule
